# Factors associated with family, school and behavioral characteristics on sexual initiation: A gender analysis for Brazilian adolescents

**DOI:** 10.1371/journal.pone.0208542

**Published:** 2018-12-10

**Authors:** Marco Túlio Aniceto França, Gustavo Saraiva Frio

**Affiliations:** Departament of Economics, Pontifical Catholic University of Rio Grande do Sul (PUCRS), Porto Alegre, Brazil; University of Westminster, UNITED KINGDOM

## Abstract

Adolescence is a period of transition between childhood and adulthood. The article aims to study the individual, family and school characteristics of adolescents beginning their sexual lives. The database we used was the 2015 PeNSE (National Adolescent School-based Health Survey) and the methodology was the survival analysis. The results show that boys initiate sexual activity before girls and risky behaviors associated with the use of licit and illicit drugs increase the chance of having early sex. In addition, this work highlights the importance of parental practices in reducing the chances of beginning sexual activity, as well as the presence of school workshops on the risks of pregnancy. It should be noted that intervention strategies need to be differentiated according to gender in order to increase effectiveness.

## Introduction

Adolescence is a transition period between childhood and adulthood, since there are several biological, physical, emotional, cognitive and social changes [1]. It is marked by a decreasing dependence on the family, growing importance of social relationships, and an intense period of experimentation and new experiences, which often include the onset of sexual activity.

Sexual initiation usually happens during adolescence, and this subject has been drawing attention because an early onset may result in unwanted consequences in several respects. It is also due to the presence of factors associated between the early first sexual intercourse and the establishment of behavior patterns that may last for the entire life [2,3]. Despite the greater access to information and knowledge that this population has regarding the use of condoms, not all of them have access to it nor do they seem to make regular use of it in all sexual relations [4–6]. From the early motherhood viewpoint, there is a prevalence of low birth weight in children born to pregnant women under the age of 20 and this may be due to the immature female biological system, as well as the pregnant woman’s inadequate diet, according to Roth et al. (1998)[7].

Pinho et al. (2002)[4] and Chevalier and Viitanen (2003) [8] emphasize that teenage motherhood increases the chances of school dropout, and the smaller number of study years leads to future income loss due to lower wages. Novelino (2011)[9] emphasizes the high percentage of adolescent mothers in Brazil who are not in school, between the ages of 15 and 17 and between 18 and 19. While within the age group from 15 to 17, 90% of adolescents who do not have children are in school, only 30% of adolescents with children attend school units. For the age group from 18 to 19, the percentage of school enrollment among adolescents who do not have children and those who do is 53% and 20%, respectively.

Tillmann and Comin (2016) [10] emphasize that early motherhood is highly correlated with the chances of the young woman not to study and not to work (NEET generation). The authors state that 30% of Brazilian women who do not work and do not study have children. In addition, as their age increases, the percentage of them who become mothers is close to 80%. Silva (2018) [11] shows that most youngsters of the NEET generation are women with children, corroborating what Figueiredo and Almeida (2017)[12] find: being a woman and having a child increases the chances of being part of the group that does not work and does not study. A study from OECD (2014)[13] reveals that this situation is more serious in poor regions with scarce economic opportunities. The reason, according to Aquino et al. (2003)[14], is that maternity is seen in these localities as a priority, because it changes the social status of these young people before the community where they live.

Buvinic (1998) [15] points to the existence of a social cost, especially for the woman who experiences a teenage pregnancy in an out-of-wedlock situation. This result increases the chances of forming families of single mothers and households headed by women. On average, these households have lower per capita income, leading to an unexpected poverty situation. Longo (2002) [16] mentions that this situation restricts educational opportunities, limits entry into the labor market and, therefore, weakens the mother’s and the child’s quality of life, leading to a vicious circle of poverty. Finally, Chevalier and Viitanen (2003) [8] argue that programs that prevent unplanned pregnancy would reduce the need for public income support and effects associated with social exclusion.

Wellings et al. (2006) [17] show that, although there is a growth in the number of boys who have already started their sexual life in Brazil, the same is not observed among girls. Oliveira-Campos et al. (2013) [18], using the 2009 PeNSE (National Survey of School Health), point out that among Brazilian adolescents under the age of 14 and who stated that they had started sexual activity, 35% had the first sexual intercourse when they were 12 years old or younger. This behavior was different between boys and girls, being 42.3% and 19.7%, respectively, among both genders. As was mentioned before, it is important to investigate the factors associated with the onset of sexual activity between boys and girls. The knowledge of sexual behaviors is important for the development of public policies and thus contributes to debunking possible myths. In addition, this period is important because one of the possible consequences of having early sexual intercourse goes beyond the unwanted pregnancy, since there are also sexually transmitted infections, especially HPV (human papillomavirus) in women [19,20]. According to Kerr et al. (2018) [21], the cases of sexually transmitted diseases such as AIDS among men have increased considerably in the last 10 years in Brazil, in the age groups from 15 to 19 and from 20 to 24.

The research strategy chosen to verify the variables that affect the probability of having the first sexual intercourse in Brazil will be the Survival Analysis of Cox Proportional Hazards. The sample consists of individuals who are in school and the data is from the 2015 PeNSE (National School Health Survey). Thus, it will be possible to understand the factors that lead adolescents to initiate sexual activity. The chosen methodology is similar to that employed by Lammers et al. (2000) [22] who used the Cox survival model to understand which variables affect the chance of having early sex in the United States.

The evidence pointed out by the log-rank test and the Kaplan-Meier model show that girls usually begin their sexual lives later than boys. The results indicate that health-risk behaviors (such as tobacco, illicit drugs and alcohol consumption) increase the chances of initiating sexual life early. However, living with the parents/guardians, in addition to an active participation on their part in the adolescent's life, are important elements for reducing the probability of early onset sexual activity for girls. However, no similar effect is observed in boys.

The results at school level point to different effects on the age of the first sexual intercourse since, while information about obtaining condoms increases the chances of adolescents initiating early sexual life, orientations regarding the risks of pregnancy affect girls, reducing the chances of starting sexual activity early. However, such measures would not affect boys.

This work is the first to verify, at the national level, as far as we know, the factors associated with the age of sexual initiation of Brazilian students with the use of survival models, since the texts in the literature are focused on specific cities. The results point to the importance of school as an information medium to adolescents and the need for differentiated strategies for care in early sexuality with regard to gender.

The paper has five sections, in addition to this introduction. The second section shows a background of what literature points to as predictors of sexual initiation in adolescence. The next section concerns the data sources, the description of variables and the descriptive statistics. The fourth section explains the methods we used. The results are described afterwards. Finally, the results are discussed in light of the literature and the final considerations are drawn, with suggestions of public policies aimed at reducing risk behaviors.

### Background

Boys tend to start their sex life before girls and have a greater number of partners [1,23–27]. Wellings et al. (2006)[17]indicate that, in many countries, the onset of sexual activity would not correlate with boy’s age of marriage. However, for countries where early marriage is a rule, the age of first sexual intercourse is younger for girls.

The way the youngster feels about their own body is different among boys and girls. While the way one feels about their own body becomes more valuable over time in the case of girls, boys tend to have a negative body image. However, with the onset of sexual activity, boys become more pleased with their own body, while girls become less contented [28].

Although the availability of condoms increases the chances of sexual initiation, we found that boys use more condoms than girls in the latter intercourse, while girls use, on average, more contraceptive methods [27]. Oliveira-Campos et al. (2014) [29] point out that 25% of sexually active adolescents did not use condoms, and the percentage is similar to that observed in the WHO (World Health Organization) survey, but it is lower than in the United States (39.8%).

Parental monitoring refers to the parents' knowledge about the adolescent’s activities. This attitude is shown to be effective, according to Stattin and Kerr (2000) [30], when adolescents report their activities to their parents. Skinner, Johnson and Snyder (2005) [31]divided into six the parental behaviors whose characteristics would constitute the relations of proximity versus distance; structure versus chaos; affection versus coldness; involvement versus neglect; rigidity versus permissiveness, consistent discipline versus consistent indiscipline.

The literature points out that the traditional parental arrangement–living with the father and the mother–is determinant for avoiding the early sexual activity and risky behaviors [26,32–36].

There is evidence that good communication with parents may delay the age of the first intercourse [33], therefore, low parental involvement or the young person skipping classes without permission from their parents increase the chances of early sexual initiation [37]. Rodgers (1999) [38] points out that parental monitoring is more effective than regular communication with the youngster, although both have an effect on reducing risky sexual behaviors (not using condoms, having a large number of sexual partners). Family participation is indicated as an important predictor of first sexual intercourse, since rigid parental relationships can delay the onset of sexual activity [35] or reduce the likelihood of risky sexual behavior [39].

Parental education also affects the young age of the sexual activity onset, therefore, evidence shows that the mother's education [35], as well as the parents’ education [40] are negatively associated with the age of the first sexual intercourse. Parents who are more educated have, on average, children who begin their sexual lives late, even if there is evidence, according to [24], that parents' education does not affect age. The socioeconomic level issue is also something that involves the family and positively influences the age of the first sexual intercourse. Therefore, the greater the socioeconomic level, the later the sexual life begins [22,26,41], although there are studies that point out that the level of income does not impact the age of the first sexual intercourse [24,42].

It should be noted that the family socioeconomic level depends not only on the father or mother, but also on the young person in question. The literature points out that if the person in question works, the chances of initiating a sexual life are greater [26,29,42,43], even though there is evidence in the opposite direction–people who do not work start their sexual lives earlier [24]. There is also evidence, according to Cruzeiro et al., (2010) [25], that working would not affect the number of partners, a proxy for exposure to risky sexual behaviors.

The juvenile behaviors considered risky refer mainly to the use of tobacco and alcohol–either through frequent consumption or situations that lead to drunkenness–and use of illicit drugs [22,24–26,33,34,37,40,44,45]. National and international literature converge by highlighting these behaviors as predictors of the age of first sexual intercourse, sex without the use of condoms, or even the number of sexual partners.

Another important factor pointed out in the literature is the influence of schools on sexual behaviors considered risky. The presence of dedicated teachers and the young person’s enjoyment of studying reduce the chances of the sexual act occurring early [33]. Private school students are more aware of STIs (Sexually Transmitted Infections) and are less likely to initiate their sex life early [1,23]. Risky sexual behaviors are directly associated with low education levels [43,46].

Living in urban areas or in large cities increases the likelihood of having early sexual intercourse [23,41]. Physical activity is not indicated as an element that affects the sexual behaviors of young people in Brazil [24] and neither in Europe [37]. Another important factor to avoid early initiation and risk behaviors concerns youth education. Young people with higher levels of education, according to the literature, are less likely to initiate sexual intercourse early and display risky sexual behaviors–multiple partners and not using condoms–[24–26,43,46].

## Data and methods

### Data

The data we used are from the 2015 National School Health Survey (PeNSE) and correspond to a comprehensive health survey for people who are in school, that is, children and adolescents who are between the fifth and ninth grade of both public and private Brazilian schools. IBGE (Brazilian Institute of Geography and Statistics) is the responsible for collecting the information, and the research is in its third edition. The others were carried out in the years 2009 and 2012. The questionnaire is structured in modules and allows investigating several aspects associated with school-attending adolescents’ health, such as the sociodemographic characteristics and the social and family context. It also incorporates aspects related to food, body image, physical activity, smoking, alcohol and illicit drug consumption, oral health, behavior, and sexual initiation, whether they were involved in a traffic accident, or any other type of violence (including sexual) issues, safety issues (such as going to and coming from school), and anthropometric measures [47].

According to Lammers et al. (2000) [22], it is necessary to exclude people who reported having suffered some type of sexual abuse, since the sexual act was not spontaneous. In addition, individuals who did not answer all the survey questions were excluded. Therefore, we considered a total of 87,298 observations. The estimates were also used with the weight according to the sample design.

## Methods

First, the Factor Analysis method and the extraction of the main components were used to generate a variable on the socioeconomic level. The Kaiser-Meyer-Olkin (KMO) test was also used to verify the quality of the variables’ adjustment on the main components. The Varimax rotation is used to understand how the factors correspond to the variables. The aspects used in the factor analysis are in the S1 Table.

The survival analysis was performed using nonparametric (Kaplan-Meier) and semiparametric (Cox) models. The methodology seeks to analyze the survival time of a variable *T* that is random and not negative, determining the time to the event loss. The survival function *S*(*t*) is defined as the probability that the event does not occur (the loss does not happen), within the time of analysis *t*. It should be noted that *t* is a censored variable on the right and concerns the age of the students in the sample. The study event is the beginning of adolescent sexual activity.

The survival function *S*(*t*) = Pr(*T*≥*t*) is defined as the probability that the sexual act does not occur until a certain time *t*. The cumulative distribution *F*(*t*) = 1−S(*t*) corresponds to the probability of the individual engaging in sexual activity before time *t* [48]. The function is equal to 1 when *t* = 0 and decreases to zero when it tends to infinity.

The Kaplan-Meier nonparametric estimator proposed by Kaplan and Meier (1958)[49] to estimate the survival function is defined as:

$$\hat{S}(t)=\frac{Number\;of\;students\;who\;did\;not\;initiate\;sexual\;activity\;until\;time\;t}{Total\;number\;of\;students\;in\;the\;sample}$$ (1)

where $\hat{S}(t)$ is a staircase function whose steps have $\frac{1}{n}$ lenght where n is the total number of students in the study. The number of steps corresponds to the number of losses presented within time *t*.

The next step to the Kaplan-Meier model will be the estimation of the Cox proportional hazards model. The model is flexible, since it has a parametric and a nonparametric component, in addition to allowing the incorporation of covariates. Proportionality is assumed between groups, so the loss rate function of the group composed of those who have already practiced the sexual act *λ*_1_(*t*) and the loss rate function of the group formed by those who did not practice the sexual act *λ*_0_(*t*) remains constant and is equal to $\frac{\lambda_{1}(t)}{\lambda_{0}(t)}=K$.

## Results

Table 1 presents the variables used in the models mentioned in the section above, with the description and descriptive statistics divided into two groups: those who had and did not have coitus. It should be noted that all means are statistically different between groups. It is noteworthy that 29% of the sample had already engaged in the sexual act, these with average age of 13.2 at the time of their first sexual intercourse. Of the people who had already experienced their sexual debut, 63% are boys.

**Table 1:** 

		Did not perform the sexual act		Performed the sexual act		
Variable	Description	Mean	Standard Deviation	Mean	Standard Deviation	t
*Students’ Characteristics*						
Did not perform the sexual act	1 = person has not had sexual intercourse. 0 = c/c	0.71	0.45	-	-	-
Age of first sexual intercourse	Age of the first time they had sex	-	-	13.18	1.73	-
Age	Age at the time of the research	14.08	0.88	14.8	1.18	*
Male	1 = male 0 = c/c	0.42	0.49	0.63	0.48	*
Lives with the mother	1 = same residence as the mother. 0 = c/c	0.92	0.28	0.85	0.35	*
Lives with the father	1 = same residence as the father. 0 = c/c	0.65	0.48	0.55	0.5	*
Lives with the parents	1 = same residence as the parents. 0 = c/c	0.61	0.49	0.48	0.5	*
Caucasian ethnicity	1 = white. 0 = c/c	0.38	0.48	0.31	0.46	*
Black ethnicity	1 = black. 0 = c/c	0.12	0.32	0.17	0.37	*
East Asian ethnicity	1 = East Asian. 0 = c/c	0.04	0.2	0.03	0.18	*
Mixed ethnicity	1 = brown 0 = c/c	0.43	0.49	0.46	0.5	*
Amerindians ethnicity	1 = indigenous 0 = c/c	0.03	0.17	0.03	0.18	*
Mother’s education ^(1)^	Mother’s education	5.14	2.38	4.85	2.49	*
Education	Years of study	9.987	0.17	9.991	0.13	*
Employment	1 = is employed 0 = c/c	0.10	0.29	0.23	0.42	*
Toilets in the house	Number of toilets in the house	1.44	1.27	1.37	0.74	*
Socioeconomic Level	Socioeconomic level (estimated via Factor Analysis)	-0.002	1.0	-0.18	1.01	*
*Habits of the student or relatives*						
Has already smoked	1 = has already smoked. 0 = c/c	0.11	0.31	0.39	0.49	*
Smoked 30 days ^(2)^	How many days they have smoked in the last 30 days	0.15	0.52	0.73	1.32	*
Mother smokes	1 = the mother smokes. 0 = c/c	0.07	0.26	0.11	0.31	*
Father smokes	1 = the father smokes. 0 = c/c	0.13	0.33	0.16	0.36	*
Has already drank alcohol	1 = has already drank alcohol 0 = c/c	0.45	0.5	0.79	0.41	*
Drank alcohol 30 days ^(3)^	How many days they have drank alcohol in the last 30 days	0.71	1.01	1.69	1.56	*
Heavy drinking ^(4)^	How many times they had a heavy drinking episode	0.63	0.87	1.59	1.35	*
Drug use	1 = has already used illicit drugs. 0 = c/c	0.04	0.19	0.23	0.42	*
Drugs 30 days ^(4)^	How many days they have used drugs in the last 30 days	0.06	0.36	0.48	1.09	*
*Family monitoring habits*						
Skipped class without permission ^(5)^	Days they have skipped class without the parent’s permission	1.26	0.65	1.57	0.96	*
Parents knew what they were doing ^(6)^	When the parents knew what the youngster was doing	3.93	1.28	3.42	1.41	*
Parents checked the homework ^(6)^	When the parents have checked the homework	2.85	1.43	2.72	1.47	*
*School’s characteristics*						
Capital city	1 = school is located in a capital city. 0 = c/c	0.23	0.42	0.22	0.42	*
Urban	1 = school is located in urban area. 0 = c/c	0.92	0.27	0.91	0.29	*
Private school	1 = administrative dependence: private school. 0 = c/c	0.17	0.38	0.08	0.27	*
Full Time	1 = studies full time. 0 = c/c	0.21	0.40	0.25	0.43	*
*School activities*						
Pregnancy orientation	1 = has received orientation from the school on pregnancy. 0 = c/c	0.85	0.36	0.82	0.38	*
Condom orientation	1 = has received orientation from the school on obtaining a condom. 0 = c/c	0.75	0.44	0.79	0.41	*
STI orientation	1 = has received orientation from the school on STIs. 0 = c/c	0.91	0.29	0.89	0.31	*

^(1)^1 = did not study, 2 = did not finish elementary school, 3 = finished elementary school, 4 = did not finish high school, 5 = finished high school, 6 = did not finish undergraduate school e 7 = finished undergraduate school.

^(2)^1 = No day, 2 = 1 or 2 days, 3 = 3 to 5 days, 4 = 6 to 9 days, 5 = 10 to 19 days, 6 = 20 to 29 days, 7 = every day

^(3)^1 = No heavy drinking, 2 = 1 or 2 times, 3 = 3 to 5 times, 4 = 6 to 9 times, 5 = 10 or more times.

^(4)^1 = Did not use, 2 = 1 or 2 times, 3 = 3 to 5 times, 4 = 6 to 9 times, 5 = 10 or more times.

^(5)^1 = No day, 2 = 1 or 2 days, 3 = 3 to 5 days, 4 = 6 to 9 days, 5 = 10 or more days.

^(6)^1 = Never, 2 = Rarely, 3 = Sometimes, 4 = Most of the time, 5 = Always.

* t-test significant at 1%.

People who have had their sexual intercourse, in comparison to others, are older, black or brown (mixed-race), have a few more education years and have a greater presence in the labor market. As for the habits of these people and their relatives, it is verified that the people who have already had their first sexual intercourse have higher means of risky behaviors, as well as their relatives. Parents are more aware of the homework of young people who have already had their sexual debut and these young people on average skip classes more often without parental permission. There is a greater percentage of young people who study full-time and have received orientation on the use of condoms among young people who have already engaged in sexual intercourse than among young people who have not had it.

Of the characteristics of young people who did not have intercourse, they are usually women, living with their father, mother or both. People are usually white or East Asian, with mothers having higher education, on average, than the mothers of people who have had sex. Socioeconomic variables are also higher for people who did not engage in sexual intercourse, on average. Young people who have not yet had sexual activity are more concentrated in urban areas, capitals and private schools, and have received, on average, more orientation on condom use and pregnancy in their schools.

**Fig 1:**
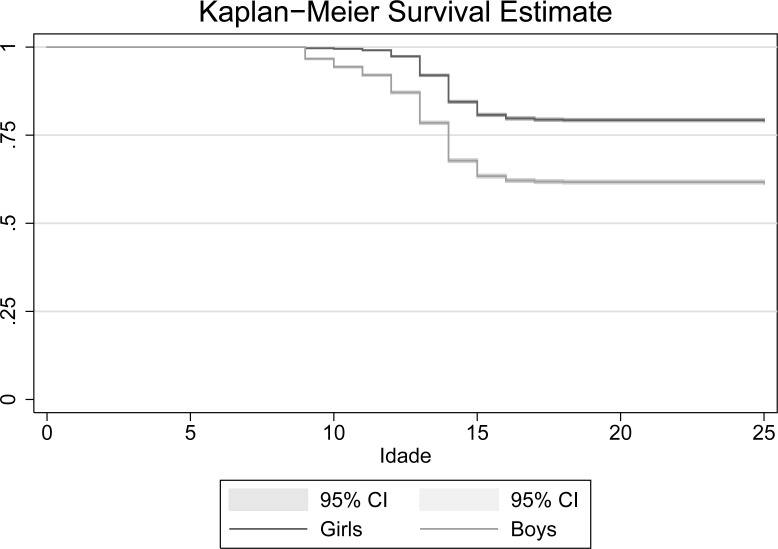
Kaplan-Meier survival model. Source: prepared by the authors.

The log-rank test shows significant difference at 1% between boys and girls from the survival rates. Fig 1 shows the difference between boys and girls in the age of first sexual activity. At the first age of the sample, 9 years, there is a location shift of boys and at age 14 this difference increases. The average survival chance of girls is greater than 75%, while the boys' survival difference is about 60%.

Table 2 presents the results of the Cox hazard model in Odds-Ratio. It should be noted first of all that living in a capital city has no effect on the age of the first sexual intercourse, nor does the school orientation on sexually transmitted infections (STIs). The use of tobacco by the father, living with both parents, having used drugs in the last 30 days and being of East Asian ethnicity also have no effects for both sexes.

**Table 2:** 

	(1)		(2)	
	Boys		Girls	
Capital city	1.025		1.042	
	(0.03)		(0.04)	
Urban	0.941		0.871	***
	(0.04)		(0.05)	
Private school	0.635	***	0.610	***
	(0.03)		(0.04)	
Pregnancy orientation	1.013		0.680	***
	(0.04)		(0.04)	
Condom orientation	1.155	***	1.223	***
	(0.04)		0.06	
STI orientation	0.998		1.028	
	(0.05)		0.07	
Full Time	1.118	***	1.076	*
	(0.03)		(0.05)	
Lives with the mother	0.949		0.741	***
	(0.05)		(0.04)	
Lives with the father	0.992		0.789	***
	(0.07)		(0.07)	
Lives with both parents	0.907		0.975	
	(0.07)		(0.09)	
Mother’s education	0.990	*	0.957	***
	(0.01)		(0.01)	
Education	1.054		1.186	
	(0.06)		(0.14)	
Employment	1.345	***	1.373	***
	(0.04)		(0.07)	
Has already smoked	1.446	***	1.664	***
	(0.06)		(0.09)	
Has smoked for 30 days	0.957	***	0.988	
	(0.01)		(0.02)	
Mother smokes	1.086	*	1.105	*
	(0.05)		(0.06)	
Father smokes	1.062		1.078	
	(0.04)		(0.05)	
Has already drank alcohol	1.692	***	1.931	***
	(0.07)		(0.11)	
Has drank alcohol for 30 days	1.115	***	1.062	***
	(0.01)		(0.02)	
Heavy drinking	1.128	***	1.221	***
	(0.02)		(0.02)	
Drug use	1.302	***	1.767	***
	(0.08)		(0.14)	
Drug use for 30 days	1.033		1.030	
	(0.02)		(0.04)	
Black	1.254	***	1.115	*
	(0.05)		(0.07)	
East Asian ethnicity	0.988		0.977	
	(0.07)		(0.08)	
Brown (mixed race)	1.168	***	1.093	*
	(0.04)		(0.05)	
Amerindians ethnicity	1.199	***	1.251	**
	(0.08)		(0.12)	
Days when skipped class without permission	1.099	***	1.122	***
	(0.02)		(0.02)	
Parents knew what they were doing	0.933	***	0.951	***
	(0.01)		(0.01)	
Parents checked the homework	1.045	***	1.007	
	(0.01)		(0.01)	
Toilets in the house	1.059	***	0.914	***
	(0.02)		(0.03)	
Socioeconomic Level	0.993		0.914	***
	(0.02)		(0.02)	
F Test	78.39		78.32	
P-Value	0.000		0.000	
Observations	41,871		45,238	
Population	1,085,300		1,164,685	

The variables that increase the likelihood of boys starting their sexual lives earlier are orientation on condom use, full-time study, employment, use of alcohol, smoking, and having smoked in the last 30 days. The amount of heavy drinking and use of alcohol in the last 30 days are also positively correlated. Black, amerindians and brown (mixed-race) are more likely to have an early start than self-declared white boys. Higher income, more days skipping class without parental consent, and parents checking their homework increase the chances of boys making their sexual debut. Among the variables that reduce the chance of early onset for boys, we have: being in private school, the mother's education, having used tobacco in the last 30 days and the parents knowing what the boy was doing.

For girls, studying full-time, working, risky behavior variables, being black, amerindians and brown (mixed-race) and skipping class without parental permission are factors associated with early onset. Factors that reduce the chances of girls having early sexual debut are: parental control, income level, living with the father or mother, being in urban areas, private school and orientation on pregnancy.

## Discussion

This paper sought to understand the reasons that lead adolescents to initiate sexual life. In general, there are no major differences in the characteristics of the risks of early sexual intercourse when comparing boys and girls, however, some results draw attention. First, although there are no significant differences in control variables, boys initiate sexual life earlier [27,50]^.^ The result is not exclusive to Brazil, and can be observed in other countries such as the United States [51]. The difference between boys and girls can be explained by behaviors rooted in men, who need to constantly prove their sexuality through the number of partners, while women do not need to prove their sexuality, in addition to usually seeking affective relationships [52,53]. The boy’s first sexual intercourse seems to be a rite of passage that reveals a transition into adulthood, while girls seek a more stable first love experience [26,29,54].

The literature also finds that sexual activity is linked to paid work outside the household [42,43,55]. Oliveira-Campos et al. (2014) [29] and Staff et al. (2012)[56] emphasize that this may be due to the increased maturity that this activity imposes on assuming adult roles. Consequently, early working life would increase the chances of forming a new family, dropping out of school, and displaying unhealthy behaviors. The adolescent’s education is also indicated as a determining factor for the age of sexual activity onset, as well as to avoid risky sexual behaviors [42,57], but our results indicate that education is not correlated with the age of the first sexual intercourse. Aquino et al. (2003)[5] argue that poor education and low income increase the chances of an early pregnancy. The authors point out that, as education continues, especially in the lower income brackets, it reduces the chances of early motherhood and fatherhood. The socioeconomic level has different effects on boys and girls, since income increases the chances of boys engaging in sexual intercourse, while girls have smaller chances [58]

Parents play a significant role in deciding when their adolescent children will begin to engage in sexual intercourse. The presence and participation of the parents reduces the chances of the adolescents initiating sexual activity early, increasing the probability of the first sexual intercourse happening for their own interest, not for the partner’s interests [59]. In addition, the higher the mother's education, the smaller the chance that adolescents will initiate sexual activities early [58]. Moreover, the literature indicates that the family arrangement composed of father and mother is an important and essential predictor of age of first sexual intercourse and risky sexual behavior [32–34]. However, the results here show that living with both parents has no effect on the age of the first sexual intercourse [57].

The evidence indicates that there are influences of parental participation in the sexual lives of the children, even when there is an authoritarian relationship with the father [25,35,39]. The results also show that when parents knew what the adolescent was doing or when they skipped class without permission, it reduced the chances of early sexual activity. As pointed out in the literature, parental involvement in the lives of young people helps to prevent early sexual activity [37]. Authoritarian parenting styles, as well as clear communication between parents and children, point to effects on the reduction of early sexual initiation. Adolescents feel more protected and, therefore, adopt less risky behaviors. In addition, it makes the incorporation of family values and social norms by adolescents easier [60–62].

The use of licit drugs (coming from tobacco and alcohol) and illicit drugs are also positively linked to early first sexual intercourse. This type of practice is considered risky and is also associated with risky sexual practices, such as not using condoms and contraceptive methods [22,24,25]. Substance use is also associated with early initiation of sexual life [33,44,45,63]. Thus, preventive campaigns regarding the hazards of risky behaviors can be successful in reducing the chances of having early sex.

Schools are important sources of information. Condom use workshops are associated with an increase in the chances of practicing the act prematurely, regardless of gender. However, it is worth emphasizing its importance for not spreading sexually transmitted infections, although many adolescents declared not using condoms when they are with fixed partners. The use of condoms often has a marginal connotation, because within a society that values monogamous relationships, this device would be correlated with infidelity, promiscuity or disease. As the emotional relationship strengthens, Paiva et al. (2008)[3] emphasize that the use of condoms decreases as the use of birth control increases. Therefore, it would increase the chance of infection by STIs. Dallo (2011)[64] points out that episodes of early pregnancy are accompanied by more lasting affective relationships and are rarely due to casual relationships.

The lack of orientation about pregnancy increases the chance of having intercourse more frequently [65]. School is the main source of information for adolescents [66] because school orientation workshops increase students' understanding of the risks of pregnancy as well as of sexually transmitted infections [20,67]. Oliveira-Campos et. al. (2014)[29] emphasize the lack of sexual health programs in the school. In addition, DeMaria et al. (2009)[68] indicate that there would be a curricular lack of information that includes HIV protection methods and STIs. Interestingly, workshops affect only girls when it comes to postponing the onset of sexual activity, not affecting boys. This is evidence of the need to differentiate strategies according to gender, since boys would not share the risks inherent to pregnancy compared to girls.

Another important result that corroborates the literature is the fact that students come from private schools [1,23]. Oliveira-Campos et. al (2014) [29] point out that public schools are the main sites for the distribution of condoms, aside from health centers. Thus, the association between access to condoms and public school may be one of the reasons that students belonging to this administrative dependency initiate their sexual life early. In addition, the main age group that uses free condoms is between 15 and 24 years old.

### Final considerations

Risky sexual behaviors, such as early sexual initiation, are associated with economic problems, since a pregnancy can lead to school drop-out–and consequently loss of productivity when in the labor market–in addition to sexually transmitted infections (ISTs). Faced with such questions, this work was based on Cox and Kaplan-Meier survival models and data from the 2015 National School Health Survey (PeNSE) to understand the predictors of sexual initiation among Brazilian students.

The main results show that boys, on average, start their sex life before girls. Living with the father or mother and the presence of sex education workshops on teenage pregnancy are effective in reducing the risk of early sexual initiation for girls. The school's orientation on where to get a condom increases the chances of both sexes starting their sex lives earlier. The use of tobacco, alcohol and illicit drugs greatly increases the chances of young people having their sexual debut.

The results are markers for public policies aimed at young people with the goal of reducing risky sexual behavior, since this type of behavior can increase the spread of sexually transmitted infections (STIs) and early pregnancies, which can lead to maternal death or miscarriage [69,70].

For both sexes, public policies of greater control in the use of tobacco, alcohol and illicit drugs are suggested because of the close correlation between this type of behavior and the beginning of sexual activity. For girls, school policies on teenage pregnancy and parental involvement campaigns in the young woman's life seem to be the safest ways to avoid an early start in sex life.

Among the limitations, it should be noted that the database does not have the age of the girls' menarche, as pointed out in the literature [71] as an important variable in the age of the first sexual intercourse. Religion and the level of religiosity are variables widely identified as important predictors for the age of the first sexual intercourse [32,39,41,72,73], but there are no such variables in the database. Another limitation of the database is that PeNSE only includes adolescents who are in school and in specific grades, making it difficult to carry out a full analysis, since adolescents who are out of school may have different characteristics. Finally, there may be measurement error due to the possibility that respondents do not report the truth in order to meet certain social concepts regarding sexuality [23]. In this sense, Wellings et al. (2006) [17] explain that Latin American culture encourages women to bias issues related to sexual activity downwards while from the male point of view the bias is up.

## Supporting information

S1 Table Factor Analysis. **Source:** Prepared by the authors based on the 2015 PeNSE. **Note:** c/c: Otherwise.
(DOCX)
