# Supplementary material for: Factors associated with family, school and behavioral characteristics on sexual initiation: A gender analysis for Brazilian adolescents
Source: PLoS One. 2018 Dec 10;13(12):e0208542. doi: 10.1371/journal.pone.0208542 (PMC6287818; doi:10.1371/journal.pone.0208542)
Supplement: S1 Table — Source: Prepared by the authors based on the 2015 PeNSE. Note: c/c: Otherwise. (DOCX) [file pone.0208542.s001.docx]

**APPENDIX**

**Table A1:** Factor Analysis

| **Description** | ***Uniquiness*** | **KMO** | **Factor 1** |
| --- | --- | --- | --- |
| 1 = has a landline at home. 0 = c/c | 0.6658 | 0.7708 | 0.5781 |
| 1 = has a cellphone at home. 0 = c/c | 0.8379 | 0.7381 | 0.4026 |
| 1 = has a computer at home. 0 = c/c | 0.3797 | 0.6704 | 0.7876 |
| 1 = has internet access at home. 0 = c/c | 0.4472 | 0.6782 | 0.7435 |
| 1 = has a car at home. 0 = c/c | 0.5758 | 0.7568 | 0.6513 |

**Source:** Prepared by the authors based on the 2015 PeNSE.

**Note:** c/c: Otherwise.
